# Role of PBAF and Mediator kinase module in the RELA-dependent activation of *CXCL1–3* inflammation genes of the NF-kB pathway

**DOI:** 10.3389/fimmu.2026.1702928

**Published:** 2026-01-30

**Authors:** Alexey V. Feoktistov, Anton A. Grigel, Ivan A. Zolin, Darya M. Yurkina, Anna V. Tvorogova, Asya M. Azieva, Sofia G. Georgieva, Nataliya V. Soshnikova

**Affiliations:** 1 Engelhardt Institute of Molecular Biology, Russian Academy of Sciences, Moscow, Russia; 2 Institute of Gene Biology, Russian Academy of Sciences, Moscow, Russia

**Keywords:** CDK8, CXCL1-3, inflammation, Mediator, NF-kB, PBAF, TNF

## Abstract

**Introduction:**

The transcription of inflammatory genes is rapidly induced by extracellular stimuli through coordinated actions of transcription factors and large coactivator complexes. However, the mechanistic interplay between specific chromatin remodelers and kinase modules in driving the transcriptional burst of early inflammatory genes remains poorly understood. This study investigates the roles of the PBAF chromatin remodeling complex and the Mediator kinase module (MKM) in activating NF-κB-dependent CXCL1, CXCL2, and CXCL3 chemokine genes.

**Methods:**

We employed a combination of molecular and genomic techniques. Protein-protein interactions were analyzed via co-immunoprecipitation (co-IP). Transcriptional outputs of CXCL1-3 genes were measured by quantitative mRNA analysis. Chromatin immunoprecipitation (ChIP) was used to assess the occupancy of RNA polymerase II (Pol II), its elongating form (Pol II-S2P), the MKM subunit CDK8, and the PBAF complex (via its BAF200 subunit) at target gene promoters. Functional contributions of the complexes were dissected using siRNA-mediated knockdown of BAF200 (PBAF) and small-molecule inhibition of the MKM.

**Results:**

PBAF and MKM physically interact with each other and with the NF-κB subunit RELA, and both complexes additively contribute to the transcriptional activation of CXCL1-3 genes. Knockdown of the PBAF-specific subunit BAF200 resulted in the loss of the entire PBAF complex from chromatin, a reduction in total Pol II and CDK8 promoter occupancy, and consequently, impaired gene induction. In contrast, MKM inhibition did not affect PBAF recruitment but specifically reduced the level of elongating Pol II-S2P and transcriptional activation. These data indicate non-redundant, stage-specific functions.

**Discussion:**

Our results demonstrate that the PBAF complex and the Mediator kinase module regulate distinct, sequential steps in the transcription cycle of CXCL1-3 genes. PBAF is critical for the initial promoter recruitment or stabilization of the transcription machinery, while MKM primarily facilitates the transition into productive elongation. Their additive positive effect and physical interaction suggest a coordinated mechanism where PBAF establishes a permissive chromatin context, enabling subsequent MKM-dependent phosphorylation events that drive the transcriptional burst of key inflammatory chemokines.

## Introduction

Multiple cellular signaling pathways converge on chromatin, and the end effect of activating various signaling pathways is changing gene expression. The induction of inflammatory genes by transcription factors such as NF-kB depends on coactivator proteins and multisubunit complexes (1). NF-kB is a family of transcriptional activators represented by five proteins RELA, RELB, REL, P52, and P50, which bind to their target elements by heterodimerizing and promote the expression of a number of inflammatory genes. After NF-kB dimers bind their target sites, enhancers or superenhancers converge with promoters, resulting in a maximum concentration of activating cofactors, which provide a powerful and rapid burst of transcription (2).

The convergence of enhancers and promoters is provided by the Mediator complex (3). The Mediator complex consists of more than 30 subunits that form the head, middle, tail and kinase (MKM) modules (4–6). The kinase module consists of four subunits: MED12, MED13, CDK8/CDK19, and cyclin C (7, 8). The Kinase module is stable and is able to exist as an independent entity (9, 10). By binding to the Mediator complex, MKM prevents the PIC complex from associating with the Mediator (11–13). Furthermore, the involvement of transcriptional activators leads to the substitution of MKM for MED26 and TFIIH, the restructuring of the Mediator-PIC complex, and subsequent activation of transcription (14–19). During activation, MKM phosphorylates transcriptional activators, a number of subunits of coactivator complexes, and some subunits of Mediator, as well as its kinase function contributes to the stabilization of MED12 and MED13 (20–22). Inhibition of kinase CDK8/19 functions or disruption of MED12 negatively affects the functions of enhancers and disrupts the gene transcription activation program (23).

Another significant event occurs during the activation of inflammatory genes: a change in the nucleosome density of promoters and enhancers and the involvement of remodeling complexes of the SWI/SNF family (24–26). SWI/SNF family complexes are divided into three types cBAF, ncBAF, and PBAF depending on the structure of the chromatin binding module, which largely determines the specificity of binding of these complexes to target loci (26, 27). cBAF, including ARID1a/b and DPF1/2/3 specific subunits, ncBAF, including GLTSCR1 and BRD9 subunits, and PBAF, including BAF200, BAF180, BRD7, and PHF10 are localized mainly on enhancers formed at the time of activation of the inflammatory response of the cell to a stimulus (23). The remodeling activity of the main ATPase BRG1 and BRM of SWI/SNF complexes promotes the maintenance of chromatin in promoter and enhancer regions in an open state (23, 28) and the binding of activating TF to these loci (29). Remodeling complexes interact with polymerase II and contribute to the release of pausing (30), which is characteristic of most inflammatory genes in the corresponding cells (31–33).

Mediator and SWI/SNF complexes are important in the coactivation of NF-kB- dependent transcription (34–36). However, not so much is known about their interplay in transcription activation: their interactions and mutual effect on each other’s recruitment. It was previously shown that in mouse intestinal cells MCM interacts with and phosphorylates the ARID1a subunit of the BAF complex, and CDK8/19 knockout leads to depletion of ARID1a-CDK8/19 dependent enhancers (37).

In this study, we investigated the mutual effect of Mediator and PBAF complexes in the process of TNF-a dependent activation using a cluster of *CXCL1–3* chemokine genes as a model of primary activated genes. *CXCL1, CXCL2*, and *CXCL3* belong to the CXC chemokine family and play a key role in inflammation and the immune response (38). The main function of these chemokines is the recruitment of neutrophils into foci of infection or damage through the activation of their common CXCR2 receptor (39). In addition to participating in acute inflammation, they promote wound healing (40), angiogenesis (41), and modulation of the tumor microenvironment (42).

Dysregulation of *CXCL1–3* is associated with chronic inflammatory diseases (43, 44), autoimmune pathologies (45), and cancer progression, in particular due to increased neutrophil infiltration and metastasis (46), which makes them important biomarkers and potential targets for therapy (47). Their expression is under strict control of NF-kB (48, 49) and other pro-inflammatory transcription factors (50, 51).

We used the model of activation of the *CXCL1–3* TNF-alpha genes in HEK293T-LR cells described in the works of I. Roninson (52, 53). Using chemical and genetic inhibition of PBAF and MKM subunits, we showed that both complexes significantly contribute to the activation of *CXCL1–3* genes. The specific PBAF module interacts and colocalizes with MKM in HEK293T-LR cells, however, they function independently of each other. PBAF and MKM activate the transcription of the *CXCL1–3* genes additively, regulating different activation stages of the *CXCL1–3* genes. PBAF is necessary for the recruitment of total PolII to promoters, and MKM regulates the stage of polymerase release into elongation, which we detected by the level of PolII-S2P. Overall, our work contributes to understanding the regulation of the mechanisms of the *CXCL1–3* inflammatory genes and the relationship between important PBAF and Mediator complexes, which has not yet been investigated.

## Results

### PBAF and Mediator promote the activation of *CXCL1–3* genes

**Figure 1:**
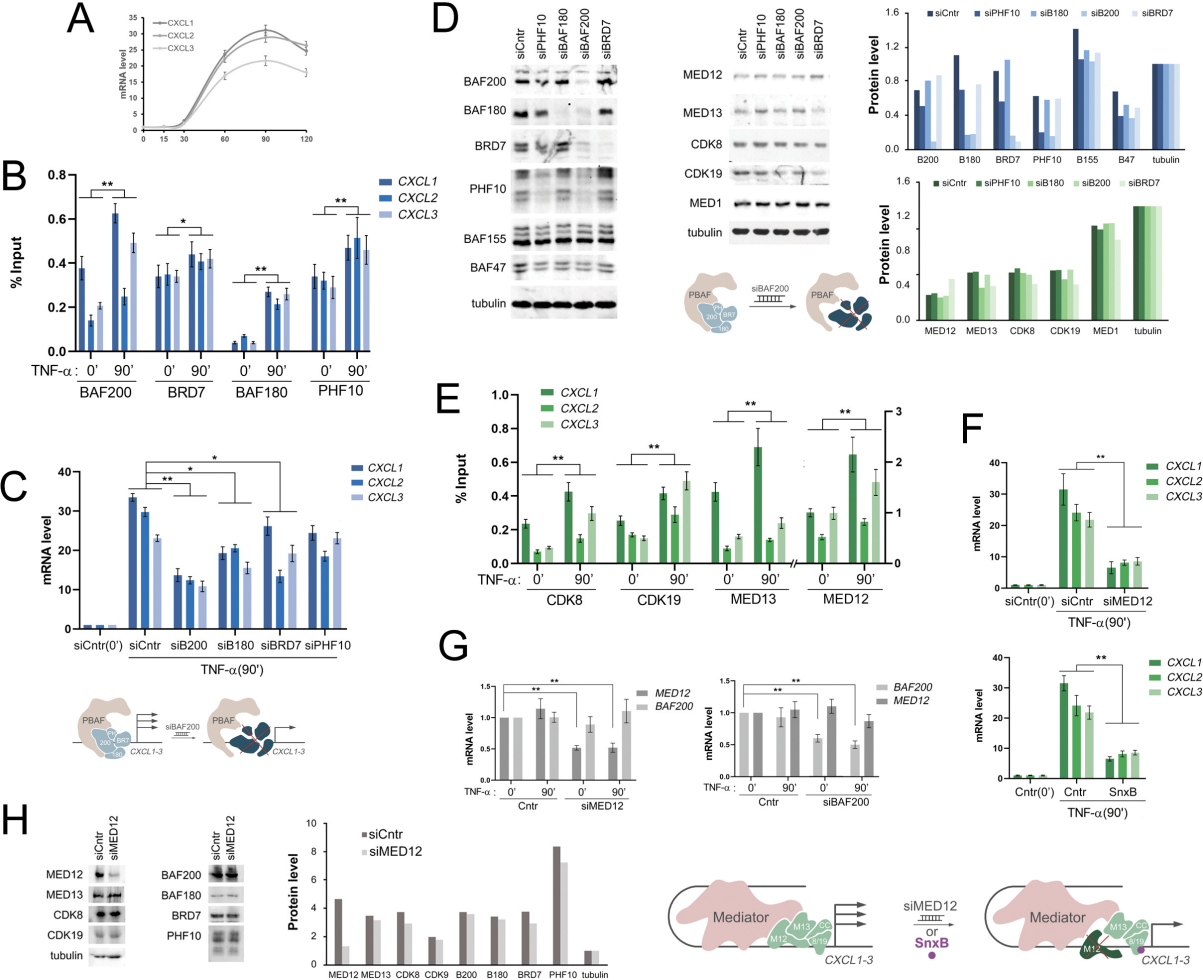
PBAF and Mediator promote the activation of CXCL1–3 genes **(A)** The level of *CXCL1–3* expression in HEK293T-LR before and after TNF-alpha (20 ng/mL) treatment. **(B)** The levels of PBAF subunits BAF200, BRD7, BAF180, and PHF10 on promoters of *CXCL1–3* genes before and following TNF-alpha treatment HEK293T-LR cells, verified by ChIP. **(C)** The level of *CXCL1–3* transcripts in the HEK293T-LR cells with knockdown of BAF200, BAF180, BRD7, and PHF10 relative to the control cells treated with siControl (siCntr) before and following TNF-alpha treatment. The level of transcripts in control cells was taken as one. The scheme below: knockdown of BAF200 results in specific PBAF module degradation and *CXCL1–3* transcription decreasing following TNF-alpha activation. **(D)** Western Blot of the control and the knockdown of PBAF subunits (siPHF10, siBAF180, siBAF200, siBRD7) HEK293T-LR cells. Tubulin was used as a loading control. The densitometry analysis of Western-Blots and the corresponding quantifications are presented on the right side. The scheme below: knockdown of BAF200 subunits lead to degradation of all PBAF specific module subunits. **(E)** The levels of MKM subunits on promoters of *CXCL1–3* genes before and following TNF-alpha treatment HEK293T-LR cells, verified by ChIP. Scale for MED12 shown on the right of the chart. **(F)** The level of *CXCL1–3* transcripts in the HEK293T-LR cells with SnxB and siMED12 treatment before and following TNF-alpha treatment. The scheme below: siMED12 or SnxB result in a decrease of *CXCL1–3* activation. **(G)** The levels of BAF200 and MED12 following their knockdown before and after TNF treatment. **(H)** Western Blot of control and siMED12 treated HEK293T-LR cells. Tubulin was used as a loading control. The densitometry analysis of Western-Blots and the corresponding quantifications are presented on the right side. Knockdown of MED12 did not affect the other MKM and PBAF subunits. In **(A, C, F, G)** experiments values represent the mean ± SD of three independent experiments and were normalized to RPLP0 expression. The Y-axis indicates mRNA fold change. In **(B, E)** values also represent the mean ± SD of three independent experiments. The Y-axis indicates % of input material. In all experiments statistically significant differences are marked as *p < 0.005 and **p < 0.0005 compared to the control (two-way ANOVA with Holm–Sidak’s multiple comparison test).

The role PBAF in secondary response is already documented in the literature (23), to study the role of PBAF and Mediator complexes in the activation of primary inflammatory genes, we used the HEK293T model line expressing the IL1R receptor (HEK293T-LR), This cell model was designed to study NF-kB dependent activation and it has been previously demonstrated to be suitable for investigating the specific role of the Mediator complex and PBAF in primary gene induction (52–55). Our objective was to dissect this specific relationship between Mediator and PBAF, which necessitated a focused analysis on well-characterized primary response genes like *CXCL1–3* cluster genes during TNF-alpha processing in model cell line HEK293T-LR. The expression of *CXCL1–3* chemokine genes during treatment with TNF-alpha cells (20 ng/mL) increased 20–30 times after 90 minutes (**Figure 1A**). 60 minutes after the addition of TNF-alpha, specific subunits of the PBAF complex BAF200, BAF180, BRD7, and PHF10 were recruited to the promoters of the *CXCL1–3* genes (**Figure 1B**).

When the subunits of a specific module of the PBAF complex were genetically inactivated using siRNA, there was a noticeable decrease in the activation of genes in this cluster (**Figure 1C**). The BAF200 knockdown led to the most dramatic decrease in the level of mRNA *CXCL1-3*. We validated the decrease in subunit expression using Western blotting and noticed that the BAF200 knockdown led to degradation of the entire PBAF module (**Figure 1D**), which could explain the critical loss of *CXCL1–3* from the BAF200 knockdown (56).

Thus, the PBAF complex is an important participant in the activation of the *CXCL1–3* cluster genes in response to TNF-alpha signaling.

We also investigated the role of another multisubunit complex, Mediator, in the activation of the *CXCL1–3* cluster genes. We were particularly interested in the role of its kinase module, as it had been previously shown that the kinase Mediator function is necessary for *CXCL1–3* activation (55). Upon the activation HEK293T cells with TNF-alpha, all subunits of the kinase module Mediator MED12, MED13, CDK8, CDK19 were recruited to the promoters of the *CXCL1–3* genes during activation (**Figure 1E**).

Knockdown of MED12 and the addition of SnxB [a compound that inactivates the kinase function of CDK8 and CDK19 (55)] to cells led to a decrease in the level of *CXCL1–3* transcription upon their activation by TNF (**Figure 1F**). At the same time, a decrease in the level of MED12 did not affect the stability of the remaining subunits of the kinase module and the expression of PBAF subunits and vice versa (**Figures 1G, H**).

Thus, we have shown that the kinase module of the Mediator complex also contributed to the activation of the inflammation genes of the *CXCL1–3* cluster.

### RELA activates the *CXCL1–3* genes cluster and interacts with PBAF and Mediator

**Figure 2:**
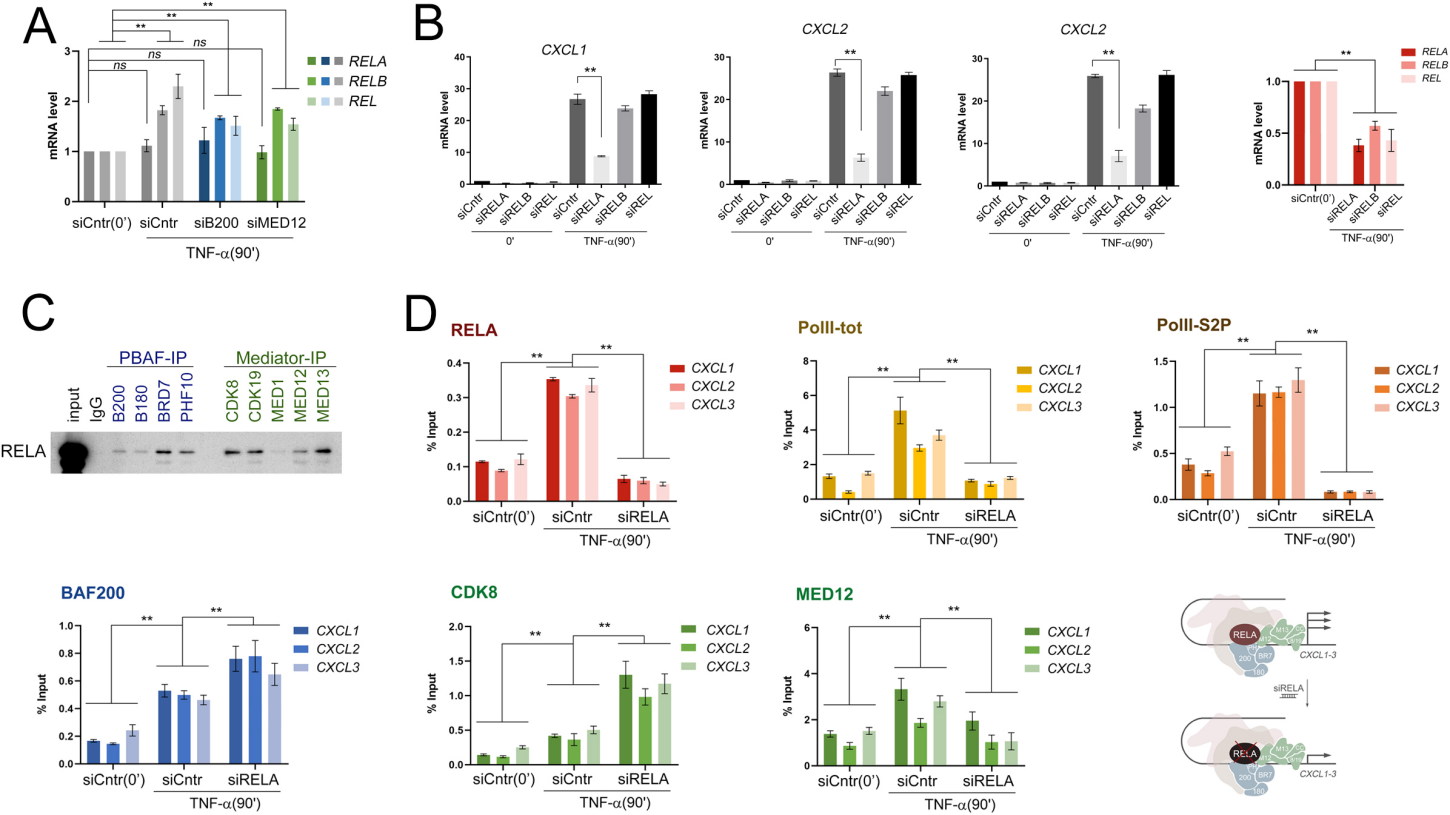
RELA interacts with PBAF and MKM modules and activates CXCL1–3 genes **(A)** Levels of mRNA REL factors following BAF200 and MED12 knockdown. *RELA, RELB*, and *REL* expressions were not dependent on BAF200 and MED12 levels. **(B)** mRNA levels of *CXCL1–3* genes following TNF-alpha treatment of HEK293T-LR. siRELA results in a more drastic decrease of *CXCL1–3* activation than siRELB or siREL. In **(A, B)** values represent the mean ± SD of three independent experiments. Experimental data was normalized to *RPLP0* expression. The Y-axis indicates mRNA fold change. **(C)** Western Blot of immunoprecipitation of RELA by antibodies against PBAF (BAF200, BAF180, BRD7, PHF10), MKM (CDK8, CDK19, MED12, MED13) modules, and MED1 subunits. **(D)** Levels of RELA, total PolII, elongated PolII (PolII-S2P), BAF200, MED12, and CDK8 on *CXCL1–3* promoters in control and siRELA treated HEK293T-LR cells following activation by TNF-alpha verified by ChIP. Y-axis represents % of input material. The scheme on the right: due to RELA knockdown, Poll, PolII-S2P, and MED12 were not recruited, but BAF200 and CDK8 were recruited following *CXCL1–3* activation. In all experiments statistically significant differences are marked as **p < 0.0005 compared to the control (two-way ANOVA with Holm–Sidak’s multiple comparison test).

It has previously been shown that *CXCL1–3* is activated by NF-kB transcription factors in response to TNF-alpha processing (50, 55). All three main activators of NF-kB are expressed in our model line HEK293T-LR: RELA, RELB, REL. We have shown that the negative effect of BAF200 and MED12 knockdowns on the activation of *CXCL1–3* genes does not occur indirectly through a decrease in the expression of RELA, RELB, and REL: we did not observe a decrease in the level of transcription of RELA, RELB, and REL after BAF200 and MED12 knockdowns either before or after TNF-alpha cell activation (**Figure 2A**). Interestingly, the RELB and REL genes themselves were activated in response to TNF-alpha treatment, unlike RELA (**Figure 2A**).

To determine which of the NF-kB factors played a major role in the activation of the NF-kB *CXCL1–3* genes, we performed a knockdown of RELA, RELB, and REL and further treated the cells with TNF-alpha. Knockdown of RELA led to a decrease in activation of *CXCL1–3* genes, more than RELB and REL (**Figure 2B**). That means, from all of the NF-kB factors, RELA plays the most prominent role in the activation of the *CXCL1–3* genes.

At the moment, there is no definite consensus in the literature on whether transcriptional coactivator complexes are recruited by activator proteins. However, the effect of the recruitment of NF-kB factors on the nucleosomal density on promoters and enhancers has been shown (24). We tested whether RELA played a role in the recruitment of PBAF and MKM subunits during the activation of our model genes. RELA was precipitated by antibodies against specific PBAF subunits and kinase module subunits from HEK293T-LR lysates (**Figure 2C**). We determined the levels of BAF200, CDK8, MED12, and total Pol and PolII-S2P elongating polymerase by chromatin immunoprecipitation, which determined the activation of *CXCL1–3* genes in normal and RELA knockdown conditions. We observed that RELA itself was normally recruited to promoters and was not recruited during knockdown (**Figure 2D**). Also, the RELA knockdown completely blocked the increase in the level of total PolII and elongating PolII-S2P on the promoters upon activation (**Figure 2D**).

The absence of the RELA activator did not decrease the number of BAF200 and CDK8 on the *CXCL1–3* promoters but led to an increase in their occupancy (**Figure 2D**). This growth could be explained by the fact that the absence of RELA does not facilitate pausing release, which causes rearrangement of transcription complexes, thus weakening association of their subunits with chromatin. However, the level of MED12 on the promoters decreased twofold in knockdown compared with the control, which could be explained by the dynamic structure of the MKM and the heterogeneity of the functions of the subunits in the kinase module (57). Thus, the recruitment of RELA to the promoters plays a crucial role in the activation of NF-kB genes but does not affect the involvement of the PBAF module and CDK8 kinase on promoters.

### PBAF and MKM coprecipitate each other, co-localize and have positive additional effect on *CXCL1–3* activation

**Figure 3:**
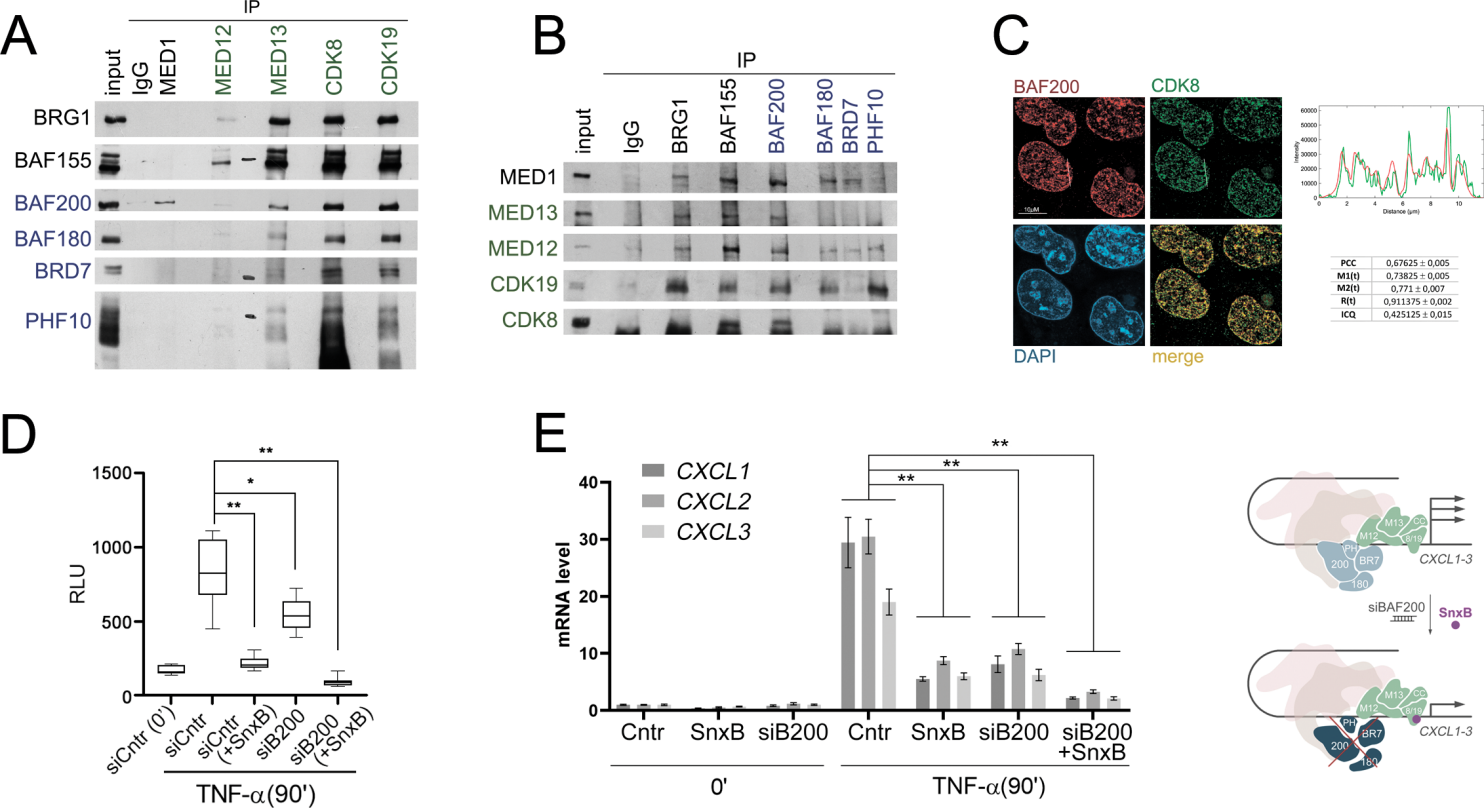
PBAF and MKM have positive additional effect on *CXCL1–3* activation **(A)** Western Blot of PBAF specific (BAF200, BAF180, BRD7, PHF10) and core (BAF155, BRG1) subunits precipitation by antibodies against MKM (MED12, MED13, CDK8 and CDK19) and MED1 from HEK293T-LR cell lysates and **(B)** vice versa. IgG was used as a control of unspecific precipitation. **(C)** Immunostaining BAF200 and CDK8 in HEK293T-LR cells. Right: plot profile across the white line of red (BAF200) and green (CDK8) signals. Below: table with Pearson’s Correlation Coefficient (PCC), thresholded Manders’ Colocalization Coefficients (M1(t) and M2(t)), Li’s Intensity Correlation Quotient (ICQ), Thresholded Overlap Coefficient (R(t)) and Li’s Intensity Correlation Quotient (ICQ) support a positive correlation between the two proteins. **(D)** Intensity of Luciferase activity (measured in relative luciferase units) in HEK293T-LR cells knockdowned with siControl (siCntr) or siBAF200 and treated or not by SnxB following TNF-alpha activation. **(E)** RNA level of *CXCL1–3* in HEK293T-LR cells in treated with siBAF200, SnxB or both before and after TNF-alpha activation. Experimental values represent the mean ± SD of three independent experiments and were normalized to *RPLP0* expression. The Y-axis indicates mRNA fold change. Scheme on the right: due to BAF200 knockdown and SnxB treatment *CXCL1–3* activation level was lower than influence of siBAF200 and SnxB separately. In **(D, E)** statistically significant differences are marked as *p < 0.005 and **p < 0.0005 compared to the control (two-way ANOVA with Holm–Sidak’s multiple comparison test).

Next, we studied the possible mutual influence of PBAF and MKM in the activation of NF-kB dependent genes. We performed the mutual co-precipitation of the subunits of the complexes with specific antibodies. Antibodies against specific subunits of the PBAF module co-precipitated the subunits of MKM from HEK293T-LR cells nuclear extract treated with DNAse and vice versa (**Figures 3A, B**). Immunostaining of BAF200 and CDK8 in HEK293T-LR cells was also performed (**Figure 3C**).

Colocalization of BAF200 and CDK8 was assessed using confocal microscopy.

The spatial correlation between BAF200 and CDK8 was assessed with the plot profile of interest (ROI) and illustrated the intensity variations of both fluorescence signals across the selected line, showing a high degree of overlap between the peaks of BAF200 (red) and CDK8 (green). Notably, the intensity fluctuations of the two channels followed a similar pattern along the ROI, with several regions exhibiting synchronous increases and decreases in fluorescence (**Figure 3C**).

Pearson’s Correlation Coefficient (PCC) analysis yielded a mean value of 0.67 ± 0.005, suggesting a correlation between the fluorescence signals of BAF200 and CDK8. Thresholded Manders’ Colocalization Coefficients (M1(t) and M2(t) coefficients) revealed that 74% of the BAF200 signal colocalized with CDK8 (M1(t) = 0.73 ± 0.005), while 77% of the CDK8 signal colocalized with BAF200 (M2(t) = 0.77 ± 0.007). Additionally, Thresholded Overlap Coefficient (R(t)) calculated as the product of the normalized intensities of two signals (0,91 ± 0,002) and Li’s Intensity Correlation Quotient (ICQ) measured the dependency between two signals, based on intensity distribution (0.43 ± 0.015) and was calculated to assess signal dependency, further supported a correlation between the two proteins.

To study the indirect or additive effect of PBAF and MKM on the activation of *CXCL1–3* genes, we inactivated the PBAF module and the kinase function of Mediator separately and together. HEK293T-LR cells contained an embedded luciferase reporter gene. We analyzed the activation of the reporter during the BAF200 knockdown and SnxB treatment. Knockdown for 48 hours and SnxB treatment for 15 minutes before activation together reduced LR luminescence more than the destruction of the PBAF module or inactivation of kinase function separately (**Figure 3D**). The effect of BAF200 and SnxB knockdown on the activation of endogenous genes of the *CXCL1–3* cluster was similar (**Figure 3E**).

Thus, the complexes interact and are localized at the same loci in cells but independently contribute to NF-kB activation.

### PBAF and KM Mediator independently regulate different steps of activation

**Figure 4:**
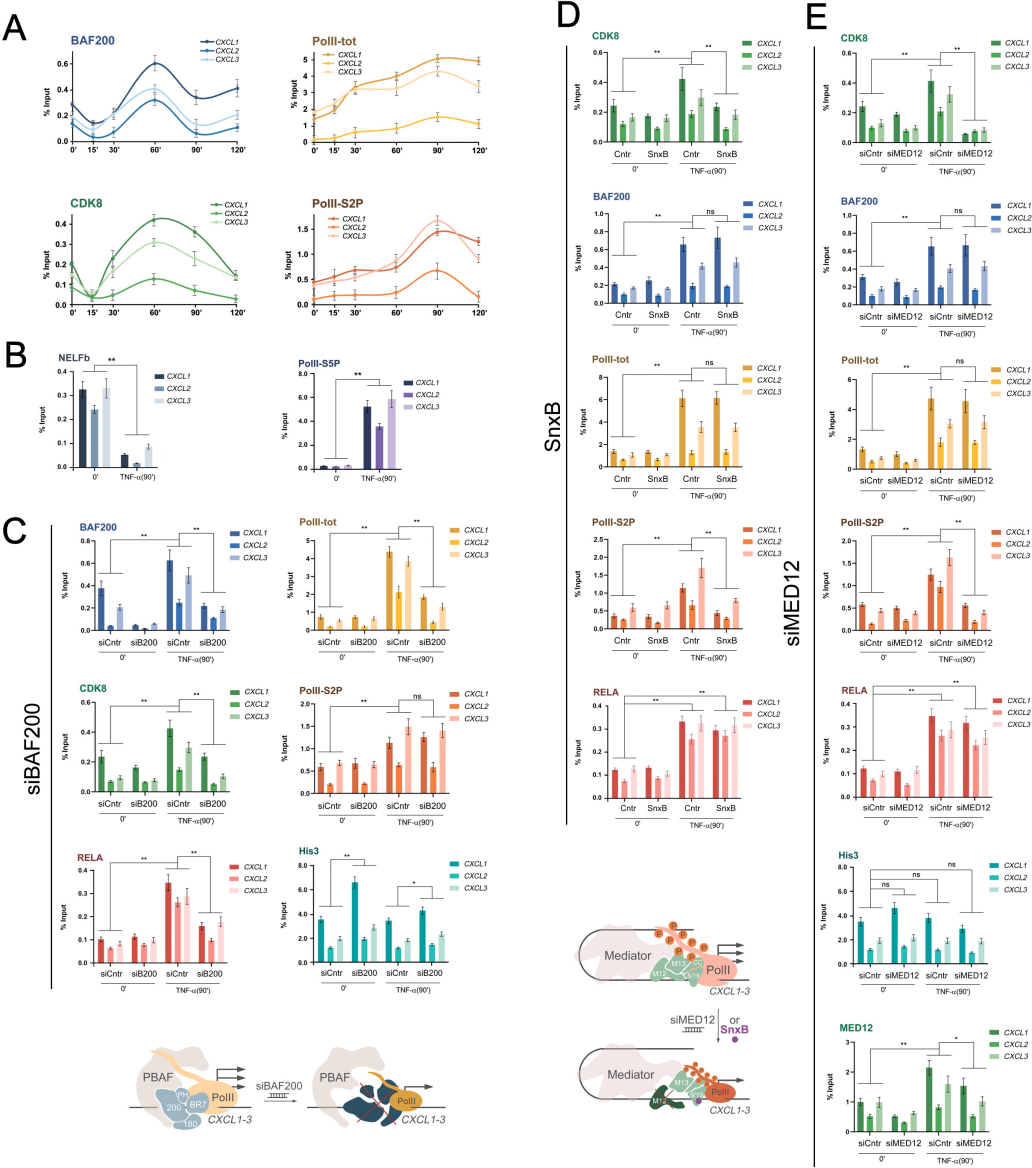
PBAF and KM Mediator regulate different steps of activation independently **(A)** Dynamics of MKM (CDK8) and PBAF (BAF200) subunits, total and S2P RNA-polymerase II on *CXCL1–3* promoters following TNF-alpha treatment of HEK293T-LR revealed by ChIP. **(B)** Dynamics of NELF (NELFb) and PolII-S5P characterize the exit of PolII from pausing, following the *CXCL1–3* activation. The recruitment of BAF200, CDK8, total RNA-polymerase II, S2P RNA-polymerase II, and RelA as well as the level of His3 on the *CXCL1–3* gene promoters upon the activation of TNF-alpha in control and knockdown by siBAF200 **(C)**, siMED12 **(E)** or treated by SnxB **(D)** HEK293T-LR cells. Values of these ChIP experiments represent the mean ± SD of three independent experiments. The Y-axis indicates % input material. Statistically significant differences are marked as *p < 0.005 and **p < 0.0005 compared to the control (two-way ANOVA with Holm–Sidak’s multiple comparison test). The schemes below show that PBAF and MKM regulate the level of Pol and Pol-S2P accordingly and independently during *CXCL1–3* activation by TNF-alpha.

The co-precipitation of complexes from nuclear extract led to a suggestion that they could influence each other’s binding to the promoters of activated genes. We studied the dynamics of PBAF and MKM promoter binding during the activation of *CXC1-3*. The kinetics of BAF200 and CDK8 recruitment during activation were similar. The level of the kinase module and the specific PBAF module decreases at the beginning and then increases above the baseline level by 60 minutes (**Figure 4A**).

A decrease in CDK8 levels in 15 minutes after activation correlated with the function of MKM. It was shown that MKM is replaced by MED26, which triggers PIC rearrangement, phosphorylation of PolII by Ser2, and the onset of active productive transcription (3). A decrease in PBAF association with the promoter immediately after the addition of TNF-alpha may also reflect the dynamic rearrangement of coactivator regulatory complexes during activation.

Thus, the dynamics of the factors of the specific PBAF and MKM modules during activation were the same, that indicates that the complexes work synchronously. Total polymerase (PolII-tot) was initially present on the *CXCL1–3* promoters, after activation the PolII-tot levels gradually increased and a maximum was observed by 90 minutes. The level of PolII-S2P elongating polymerase increased much more dynamically, and also reached a maximum by 90 minutes, which coincided with the maximum accumulation of *CXCL1–3* mRNA (**Figures 1A**, **4A**). We also analyzed the level of NELF and PolII-S5P on *CXCL1–3* promotors before and after TNF-a addition (**Figure 4B**). The dynamics of these factors validated that PolII was stalled in pausing on *CXCL1–3* promoters without activation.

Next, we examined the direct impact of factors on recruiting each other and PolII. BAF200 knockdown (degradation of the chromatin binding module of the PBAF complex) led to a decrease in BAF200 levels on *CXCL1–3* promoters before and after activation (**Figure 4C**). This reduced the amount of CDK8 and total PolII during the activation of *CXCL1-3* but did not affect the level of elongating polymerase during activation (**Figure 4C**). To study the role of the PBAF’s contribution to nucleosome density regulation we measured the His3 (H3) level at the promoter region and observed a significant increase in H3 occupancy even under basal conditions, and this trend towards a denser chromatin state was maintained, albeit to a lesser degree, following TNF-a activation. Thus, the disruption of the PBAF-specific module negatively affected the activation of NF-kB dependent genes due to the increase of nucleosome density on the promoters, which reduces the recruitment of primary PolII, CDK8, and RELA during activation. It did not affect the process of polymerase activation itself (phosphorylation by Ser2) but led to a decrease in transcription level.

MED12 knockdown and SnxB treatment displayed results identical to each other (**Figures 4D, E**). They did not affect the dynamics of His3, BAF200, total PolII, or RELA recruitment upon activation, but reduced the level of CDK8 (**Figures 4D, E**). In line with the function of CDK8 in stimulating pausing release the level of phosphorylated by Ser2 PolII (PolII-S2P) was also decreased (58, 59).

## Discussion

Chromatin remodeling is an important step in the activation of inflammatory genes (25, 60). All three SWI/SNF complexes BAF, PBAF, and GBAF were found to co-localize on the promoters and enhancers of these genes (23). While the contribution of the classical BAF complex to the activation of these genes was studied (34, 60, 61), very little is known about the contribution of PBAF. Moreover, there was evidence that PBAF did not affect the activation of primary inflammatory genes (62). Here we studied the unprecedented role of the PBAF complex as well as its relationship with MKM, polymerase, and NF-kB in the activation of NF-kB-dependent early inflammatory genes of the *CXCL1–3* cluster. We have shown that the PBAF complex and the kinase module of the Mediator complex made a significant positive contribution in activation of the *CXCL1-3*. The BAF200 knockdown, which causes the destruction of a specific PBAF module responsible for interaction with chromatin modifications, leads to a significant decrease in the level of *CXCL1–3* transcription. A decrease in transcription level was also observed with MED12 knockdown or chemical inactivation of CDK8 kinase function.

Both complexes, especially MKM and the chromatin-recognizing PBAF module, are associated with each other and with RELA, as shown by the experiments using co-immunoprecipitation. They both are recruited to the promoters of the *CXCL1–3* genes and colocalize in nucleus, which indicates their joint work in most sites of active transcription. However, PBAF and MKM are functionally independent from each other in the activation of *CXCL1–3* genes.

The dynamics of MKM and PBAF interaction with promoter were the same throughout the entire period of *CXCL1–3* gene activation. In 15 minutes after the addition of TNF-a, we observed a decrease in CDK8 and BAF200 levels on the promoters, which correlated with the release of polymerase from the pausing state (as we have shown through the decrease of NELFb levels upon *CXCL*1–3 activation), which is characteristic of inflammatory genes (33, 63). The PolII release is accompanied by withdrawal of MKM from the promoters, subsequent restructuring of PIC and recruitment of TFIIH, which phosphorylates polymerase by Ser5 and Ser2 to start the effective transcription (3). Indeed, in our experiments, the knockdown of MED12 and chemical inhibition of kinase function of MKM lowers the level of elongating polymerase PolII-S2P, that, according to the previously obtained data, disrupts the release of the polymerase from pausing (55, 64).

Degradation of the PBAF chromatin recognition module caused by the knockdown of its key subunit BAF200 leads to a decrease in CDK8 at *CXCL1–3* promoters. The BAF200 knockdown increases the H3 level, which indicates the increase of the nucleosome density, and as a consequence, lowers the level of total polymerase II on *CXCL1–3* promoters, but does not affect the elongation by PolII-S2P. These results are in line with the role of PBAF in maintenance of open chromatin during pausing (30) and characterize the *CXCL1–3* gene promoters in our HEK293T-LR model as residing in an open chromatin state, akin to the pre-configured, nucleosome-depleted architecture typical of poised immune response genes in differentiated immune cells (65). The destruction of the chromatin-binding module of PBAF complex leads to dissociation of the entire complex from chromatin and an increase in nucleosome density, which prevents binding of polymerase, CDK8, and RELA at the time of activation of *CXCL1-3* (66). Thus, the PBAF complex reduces the nucleosome density by its presence on promoters so that MKM and, in particular, CDK8 can bind to the promoter in advance of activation to prepare the molecular landscape of the promoter for subsequent transcription burst.

In our experiments, the knockdown of RELA led to a dramatic decrease in the activation of the *CXCL1–3* cluster. This indicates that the recruitment of RELA to promoters is a trigger for the S2P phosphorylation of RNA polymerase II and subsequent burst of transcription of the *CXCL1–3* genes (55). It was also shown that NF-kB factors, in particular, RELA and REL, promote chromatin remodeling of promoters and enhancers upon activation (24). The removal of these factors leads to a loss of remodeling activity and a decrease in peaks in ATAC-seq (24). However, we did not find any effect of the RELA knockdown in the attraction of the PBAF module and CDK8 kinase to promoters upon subsequent addition of TNF-alpha. It is likely that PBAF recruitment depends on other factors, such as AP-1 and STAT, which are characterized as pioneer factors in activation of TNF-alpha dependent transcription of inflammatory genes (67).

Thus, the PBAF complex and MKM of the Mediator complex play an important role in the activation of transcription of the *CXCL1–3* genes. The PBAF complex regulates nucleosome density and is responsible for preparing an opened chromatin for the binding of PolII and MKM, while MKM affects the PolII pausing release and transcription elongation of these genes.

## Methods

### Cell methods

The HEK293-IL1R-pELAM-Luc reporter cell line [HEK293T-LR originally described in PNAS publications (52, 53)] were kindly provided by Dr. Igor Roninson from the College of Pharmacy at Columbia University. Cells were cultivated in Dulbecco modified Eagle’s medium (PanEco) with 10% fetal bovine serum (HyClone) supplemented with 2 mM L-glutamine (Merck) and penicillin/streptomycin at 37°C, 5% CO2 in a humidified atmosphere. All cell lines were routinely tested for Mycoplasm contamination by DAPI staining. In experiments HEK293-IL1R-pELAM-Luc were pretreated with 5 µM Senexin B followed by stimulation with or without 20 ng/mL TNF-alpha.

Antibodies for experiments are listed in **Supplementary Table 1**.

### siRNA design and transfection

siRNAs targeting NF-κB factors, BAF200 and MED12 were designed using the RNAi Designer online tool (https://rnaidesigner.thermofisher.com/rnaiexpress/). The siRNA sequences are listed in **Supplementary Table 2**. The siRNAs were synthesized and annealed by DNA Synthesis (Russia). For transfection, siRNA was diluted to a final concentration of 100 pmol/μL in Opti-MEM reduced-serum medium (Thermo Fisher Scientific) and delivered into cells using the GenJect-39 transfection reagent (Molecta), following the manufacturer’s protocol.

### Luciferase reporter assay

HEK293-IL1R-pELAM-Luc cells were seeded into a 96-well plate and transfected with siRNA and the RenillapRL plasmid (Promega). The RenillapRL plasmid (Promega) was used for signal normalization. After twenty-four hours, the cells were harvested. Luciferase signals were measured using a single-tube assay kit (Biotium) on a Biotek Synergy 4 luminometer, following the manufacturer’s protocol. Statistical analysis was performed using one-way ANOVA. The assumptions of ANOVA were verified using the Shapiro-Wilk test (normality within each group) and Levene’s test (homogeneity of variance across groups). The data met both assumptions.

### Protein extraction and western blot analysis

HEK293T-LR were lysed in RIPA buffer (50 mM Tris-HCl (pH 7.4); 1% NP-40; 0.5% sodium deoxycholate; 0.1% SDS; 150 mM NaCl; 2 mM EDTA, supplemented with Protease Inhibitor Cocktail (PIC, Roche); Phosphatase Inhibitor Cocktail 3 (PhIC3, Sigma-Aldrich). After debris sedimentation by centrifugation at 12,000 × g for 15 minutes at 4°C protein concentrations were determined using the Qubit Protein Assay Kit (Thermo Fisher Scientific). Protein samples were mixed with 4× Laemmli buffer (200 mM Tris-HCl [pH 6.8], 4% SDS, 40% glycerol, 0.01% bromophenol blue, 100 mM DTT) at a 3:1 ratio (sample:buffer) and denatured by boiling at 95°C for 10 minutes. Equal amounts of protein (typically 20-30 μg) were separated by SDS-PAGE on 10% PAAG. Proteins were electrophoretically transferred to nitrocellulose membranes (0.45 μm pore size; Bio-Rad) using standard wet transfer protocols (Bio-Rad). Membranes were blocked with 5% non-fat dry milk (Cell Signaling Technology) in PBS containing 0.1% Tween-20 for 1 hour at room temperature with gentle agitation. Following blocking, the membranes were incubated with primary antibodies diluted in PBS containing 0.1% Tween-20 (PBST) overnight at 4°C with gentle agitation. After three washes with PBST (5 min each), the membranes were probed with horseradish peroxidase (HRP)-conjugated secondary antibodies diluted in 5% non-fat dry milk in PBST for 1 h at room temperature. Unbound antibodies were removed by three additional PBST washes (5 min each). For chemiluminescent detection, the membranes were incubated with Clarity™ Western ECL Substrate (Bio-Rad) for 5 min at room temperature. Excess substrate was carefully drained, and the membranes were immediately imaged using a Bio-Rad ChemiDoc™ MP Imaging System. Signal acquisition was performed in chemiluminescence mode with automatic exposure optimization to prevent saturation. Image analysis was conducted using Image Lab™ Software (Bio-Rad).

### Immunoprecipitation

For co-immunoprecipitation experiments, 3 × 10^6^ cells were lysed in Lysis buffer (10 mM HEPES (pH 7.9); 5 mM MgCl_2_; 0.5% Nonidet P-40; 0.45 M NaCl; 1 mM DTT supplemented with PIC (Roche), PhIC3 (Sigma-Aldrich)). The lysate was diluted 2-fold with the same buffer (omitting NaCl) and centrifuged. Cleared supernatants were incubated with 3-5 μg of target-specific antibodies and 10 μL of pre-washed MabSelect SuRe™ beads (GE Healthcare) overnight at 4°C with rotation. The Sepharose beads were washed once with IP500 buffer (25 mM Tris-HCl, pH 7.9, 5 mM MgCl_2_, 10% glycerol, 100 mM NaCl, 0.1% NP-40) and once with IP100 buffer (same composition). Proteins were eluted by boiling at 95°C for 10 min in SDS loading buffer (50 mM Tris-HCl, pH 6.8, 4% SDS, 10% glycerol, 4 mM EDTA, 0.1 M DTT, 0.1% bromophenol blue). Eluates were analyzed by Western blotting using standard protocols.

### Gene expression analysis

Total RNA was isolated from 3 × 10^6^ cells using TriReagent (MRC) according to the manufacturer’s protocol. For cDNA synthesis, 2 µg of total RNA was reverse-transcribed using oligo(dT) primers and MMLV reverse transcriptase (Thermo Fisher Scientific). Quantitative real-time PCR was performed using 5X Fast Probe qPCR Mastermix (BiolabMix) supplemented with EvaGreen. Gene-specific primers are listed in **Supplementary Table 3**. All expression values were normalized to the housekeeping gene *RPLP0*. Experiments were performed in at least three independent biological replicates, with data presented as mean ± SD. Statistical significance was determined by one-way ANOVA with Holm-Sidak’s multiple comparison test using GraphPad Prism 8.0 software, with p-values < 0.05 considered significant. ANOVA assumptions were verified using Shapiro-Wilk test for normality within groups and Levene’s test for homogeneity of variance across groups; all data met these assumptions.

### Chromatin immunoprecipitation assay

For chromatin preparation, 3 × 10^6^ cells were fixed in 10 mL of media containing 0.75% formaldehyde for 10 minutes at room temperature. The reaction was quenched by adding glycine to a final concentration of 0.125 M. After 5 minutes incubation, cells were pelleted by centrifugation at 2,500 rpm for 5 minutes and washed twice with cold PBS. Cells were resuspended in sonication buffer (50 mM Hepes-KOH [pH 7.9], 140 mM NaCl, 1 mM EDTA, 0.1% sodium deoxycholate, 0.1% SDS, 1% Triton X-100, supplemented with PIC) and sonicated using 10 cycles of 10-second pulses with 50-second intervals between pulses. The resulting DNA fragments averaged 300 bp in size (range: 100–500 bp). The lysate was centrifuged twice at 12,000 rpm for 15 minutes at 4°C. For chromatin immunoprecipitation, 3-5 μg of specific antibody or control rabbit IgG was incubated with chromatin extracts overnight at 4°C with constant rotation. MabSelect SuRe™ beads were then added and incubation continued for an additional 2 hoursBeads were sequentially washed with Sonication buffer, Buffer A (Sonication buffer containing 500 mM NaCl), Buffer B (20 mM Tris-HCl [pH 8.0], 1 mM EDTA, 250 mM LiCl, 0.5% NP-40, 0.5% sodium deoxycholate), and finally with TE buffer (50 mM Tris-HCl, 1 mM EDTA [pH 8.0]). Bound proteins were eluted using elution buffer (50 mM Tris-HCl, 1 mM EDTA, 1% SDS). DNA-protein crosslinks were reversed for both experimental samples and control inputs by incubation at 55°C for 8 hours followed by incubation at 65°C to completion. To all samples and controls, we added 12.8 μL of 5 M NaCl, 4 μL of 0.5 M EDTA, and 3 μL of Proteinase K (ThermoFisher Scientific). Prior to processing, input controls were brought to equal volume with experimental samples using Elution Buffer. Purified DNA was analyzed by quantitative real-time PCR using gene-specific primers (**Supplementary Table 4**). Experiment had at least two biological replicates. The data are presented as the mean ± SD. The normality of data was tested using the Shapiro–Wilk test, all datasets met the condition for normality (p>0.05). Two-way analysis of variance (ANOVA) followed by Holm-Sidak’s *post hoc* test for multiple comparisons was used (GraphPad Prism 8; GraphPad Software, San Diego, CA). p-value <0.05 was taken as evidence of statistical significance.

### Confocal microscopy

HEK293-IL1R-pELAM-Luc cells were grown on glass coverslips, then fixed with 4% formaldehyde. The cells were rinsed three times with TPBS, and then the samples were placed into blocking solution 1% BSA in PBS for 30 min at room temperature. The BAF200 was stained with polyclonal rabbit antibodies against BAF200 (1:40) and goat anti-rabbit Alexa Fluor ™ 633 (Life Technologies). The CDK8 was stained with polyclonal goat antibodies against CDK8 (1:40) and a donkey anti-goat Alexa Fluor ™ 488. Following washing with PBS, the coverslips were embedded in ProLong Gold (Thermo Fisher Scientific). Fluorescence images were obtained using a Leica STELLARIS 5 confocal microscope (Leica) and then analyzed using Leica confocal software (2.61.15) and processed in ImageJ 1.54f (LOCI). To collect the spectral emission of HEK293 produced by autofluorescence, a 60x (NA 1.4, oil) objective was used.

## Data Availability

The original contributions presented in the study are included in the article/**Supplementary Material**. Further inquiries can be directed to the corresponding author.
