# Supplementary material for: Role of PBAF and Mediator kinase module in the RELA-dependent activation of CXCL1–3 inflammation genes of the NF-kB pathway
Source: Front Immunol. 2026 Jan 30;17:1702928. doi: 10.3389/fimmu.2026.1702928 (PMC12901329; doi:10.3389/fimmu.2026.1702928)
Supplement: Supplementary file 1 [file DataSheet1.pdf]

Supplementary materials

Table 1. Antibodies

RelA, NF-kB p65/RelA Rabbit pAb	A2547, ABclonal Technology
Anti-RNA polymerase II CTD repeat YSPTSPS (phospho S2) antibody	Cat.№ ab5095, Abcam
RNA pol II antibody (mAb)	Cat. № 39097, Active Motif
E7 anti-beta tubulin	Cat.№ AB_2315513, Hybridoma Bank
BAF200, BAF180, BAF155, BAF47, BRD7, PHF10	Our Lab ()
CDK8, CDK19, MED1, MED12, MED13	Roninson Lab (10.1093/nar/gkad538, 10.7554/eLife.96465)
M2 anti-FLAG	

Table 2. siRNA sequences

**Name**	**Sequence 5'-3'**
siCtrl_for	GACCCGCGCCGAGGUGAAGdTdT
siCntr_rev	CUUCACCUCGGCGCGGGUCdTdT
siBAF200(h)-II_for	CAAGGGACUUCUGGCAACCAGGdTdT
siBAF200(h)-II_rev	CUGGUUGCCAGAAGUCCCUUGdTdT
siBAF200(h)-III_for	AAGGUACAUCAGGAGAAUGGdAdT
siBAF200(h)-III_rev	CCAUUCUCCUGAUGUACCUUdTdT
siMED12(h)-I_for	UCUUCGACCUCAUGGAAUAUUdCdA
siMED12(h)-I_rev	AAUAUUCCAUGAGGUCGAAGAdTdG
siMED12(h)-II_for	AGCCAGGUGCACCAGAUUGUGdAdA
siMED12(h)-II_rev	CACAAUCUGGUGCACCUGGCUdGdT
siPHF10(h)-I_for	CAGCAUUGCGCAGUGAUGAAGdTdT
siPHF10(h)-I_rev	CUUCAUCACUGCGCAAUGCUGdTdT
siPHF10(h)-II_for	AAGGUCAGUUCUUACCCAGUGdTdT
siPHF10(h)-II_rev	CACUGGGUAAGAACUGACCUUdTdT
siRELA(h)-I_for	GAUACAGACGAUCGUCACCGGdAdT
siRELA(h)-I_rev	CCGGUGACGAUCGUCUGUAUCdTdA

siRELB(h)-I_for	GAUUUGCCGAAUUAACAAGGAdAdA
siRELB(h)-I_rev	UCCUUGUUAAUUCGGCAAAUCdCdG
siRELB(h)-II_for	CGGAUUUGCCGAAUUAACAAGGdAdA
siRELB(h)-II_rev	CCUUGUUAAUUCGGCAAAUCCGdCdA
siREL(h)-I_for	AUGAGCAAUUGAGUGACUCCUdTdT
siREL(h)-I_rev	AGGAGUCACUCAAUUGCUCAUdTdT
siREL(h)-II_for	GUUGUGCAGAUAACAGCAUGAUdAdA
siREL(h)-II_rev	AUCAUGCUGUUAUCUGCACAACdTdG

Table 3. mRNA expression primers

**Name**	**Sequence 5'-3'**
RPLP0(h)_mRNA_for	ACTGGAGACAAAGTGGGAGCC
RPLP0(h)_mRNA_rev	CAGACACTGGCAACATTGCG
CXCL1(h)_mRNA_for	TAGCCACACTCAAGAATGGGC
CXCL1(h)_mRNA_rev	AACAGCCACCAGTGAGCTTCC
CXCL2(h)_mRNA_for	GCCCAAACCGAAGTCATAGC
CXCL2(h)_mRNA_rev	GCTTCCTCCTTCCTTCTGGTC
CXCL3(h)_mRNA_for	CATAGCCACACTCAAGAATGGG
CXCL3(h)_mRNA_rev	TTCAGCTCTGGTAAGGGCAGG
BAF200(h)_mRNA_for	AAGCAACAGCATCCACCAACAT
BAF200(h)_mRNA_rev	GAAAACCGCTGTCGCTGAAAA
BAF180(h)_mRNA_for	GTGTGATGAACCAAGGAGTGGC
BAF180(h)_mRNA_rev	TTGGCTGCTGTATGACAGGGG
BRD7(h)_mRNA_for	CCAGATTGCTCAGGGAACTCCA
BRD7(h)_mRNA_rev	AAGTTGTTTTCCATGACGGGGG
BAF155(h)_mRNA_for	CACCCCAGCCAGGTCAGATACC

BAF155(h)_mRNA_rev	GGAGGTTCCCTGCATCTTCCAG
PHF10(h)_mRNA_for	CCGGGAACGCATGGAAGAAAG
PHF10(h)_mRNA_rev	CACCATCACTGTCTAGAGCAGGGAG
MED12(h)_mRNA_for	GCTGCTCCTCTAACAATGGCAC
MED12(h)_mRNA_rev	TCTTCCAGGAGCAAACACTGCC
MED13(h)_mRNA_for	ACCGTCAGAGTTGGACTGTTGG
MED13(h)_mRNA_rev	CTGCTAGGAGGCATCCCAAAAG
MED1(h)_mRNA_for	GGTGTGTGTGGTAATGGATGTGC
MED1(h)_mRNA_rev	GCAATGAGGGACAGTGCTGG
CDK19(h)_mRNA_for	ATCAGCACTCCAGTTCTCGC
CDK19(h)_mRNA_rev	GAGCTCTGCTGAGACGAGGAA
CDK8(h)_mRNA_for	GCTGATAGGAAGGTGTGGCTTC
CDK8(h)_mRNA_rev	CCGAGGTAACTGAACTGGCTTC
RelA(h)_mRNA_for	TGAACCGAAACTCTGGCAGCTG
RelA(h)_mRNA_rev	CATCAGCTTGCGAAAAGGAGCC
RelB(h)_mRNA_for	TGTGGTGAGGATCTGCTTCCAG
RelB(h)_mRNA_rev	TCGGCAAATCCGCAGCTCTGAT

Table 4. ChIP primers

**Name**	**Sequence 5’-3’**
CXCL2(h) Promoter_for	CGGAGAGCCACAGAGCCC
CXCL2(h) Promoter_rev	CGCAGGAGCCGGGGATT
CXCL3(h) Promoter_for	CCCGTGCACTCTGGGACTTT
CXCL3(h) Promoter_rev	CCCCCTCACAGGCTGTATCT
CXCL1(h) Promoter_for	CTTTCCAGCCCCAACCATGC
CXCL1(h) Promoter_rev	GAGAGGAGCGGAAGAGCTGG

RAW Western Data

Fig. 1D

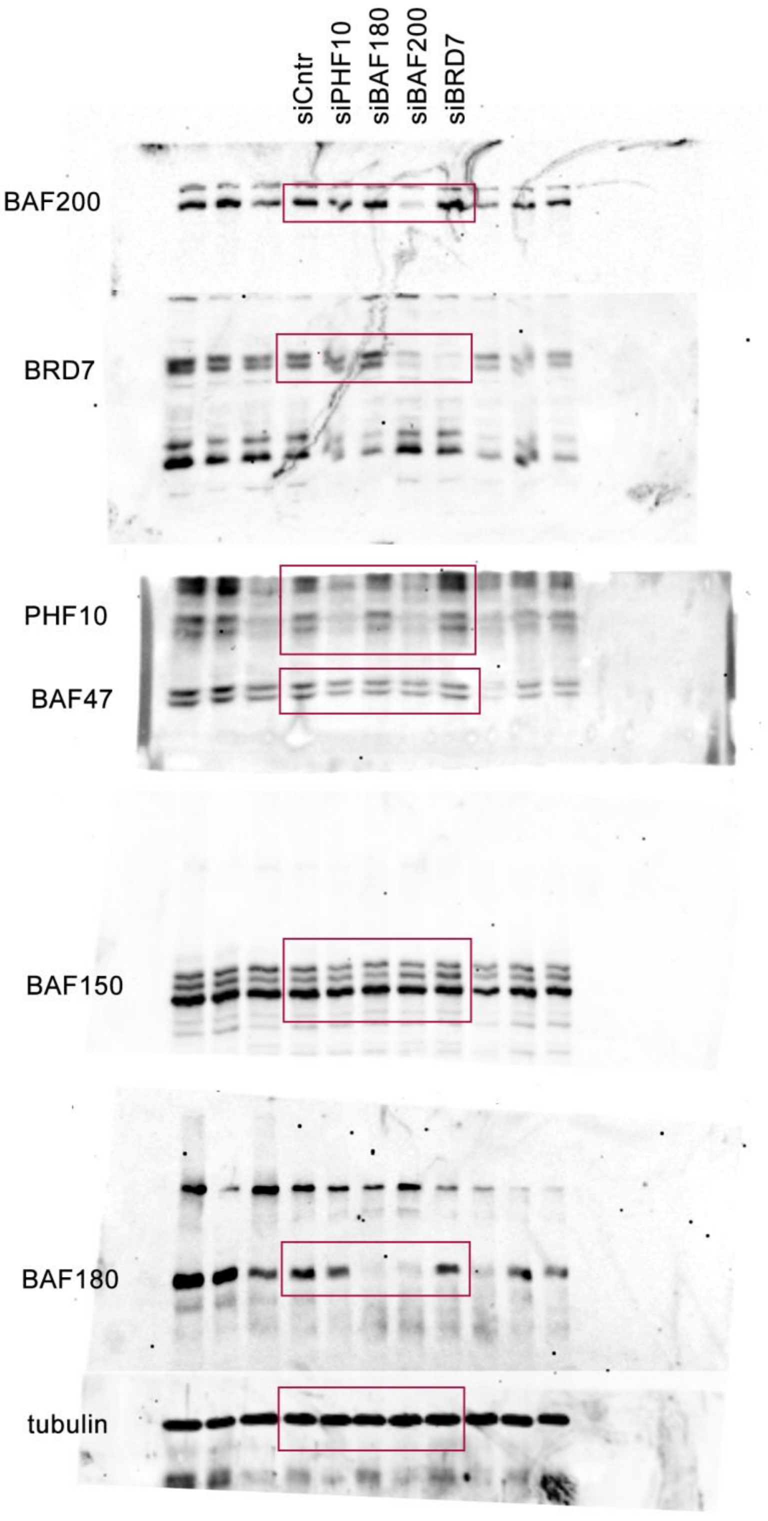

Fig. 1H

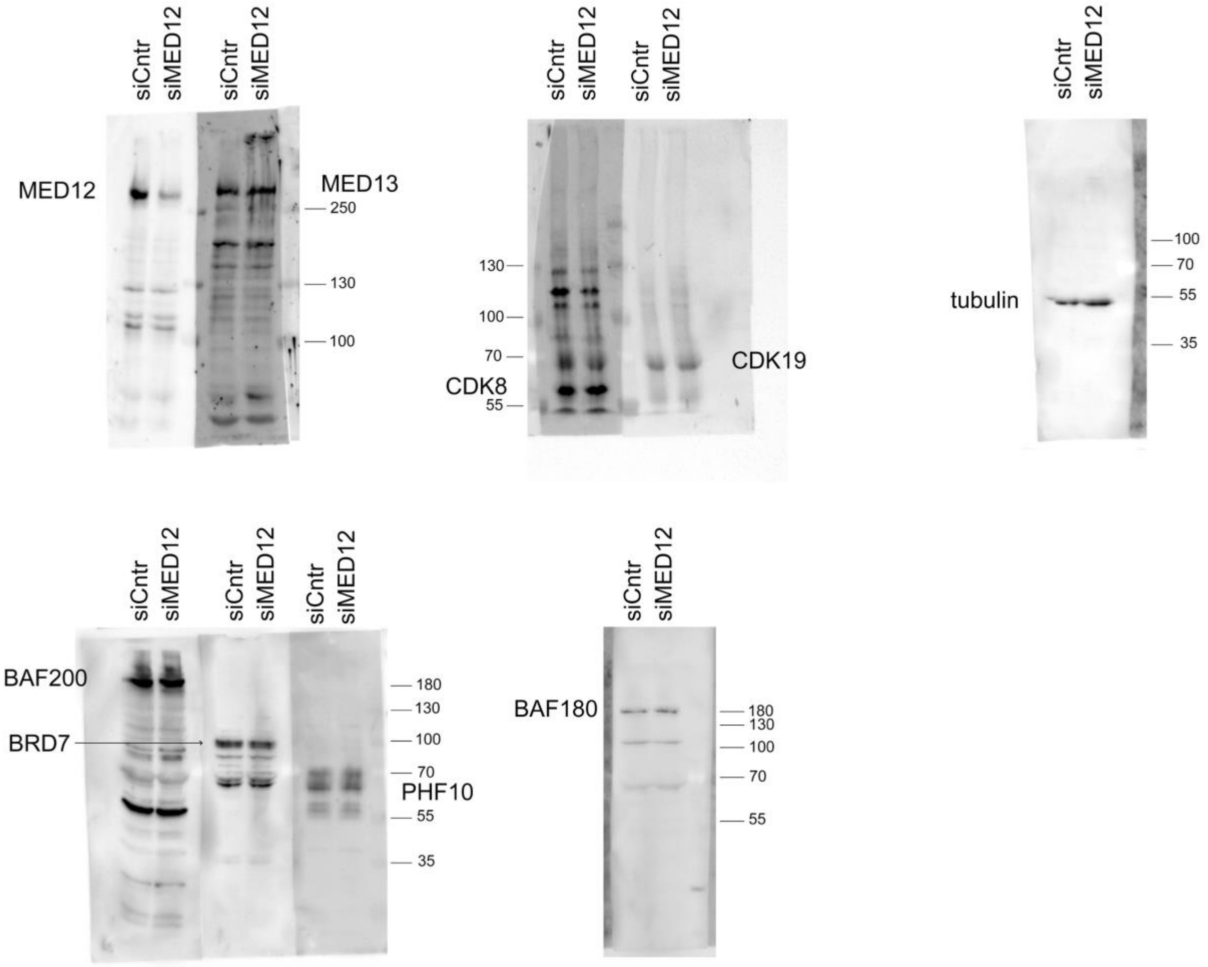
siCntr
siMED12
siCntr
siMED12
MED12
MED13
250
130
100
siCntr
siMED12
siCntr
siMED12
130
100
70
CDK8
55
CDK19
siCntr
siMED12
tubulin
100
70
55
35
siCntr
siMED12
siCntr
siMED12
siCntr
siMED12
BAF200
BRD7
180
130
100
70
PHF10
55
35
siCntr
siMED12
BAF180
180
130
100
70
55

Fig. 2C

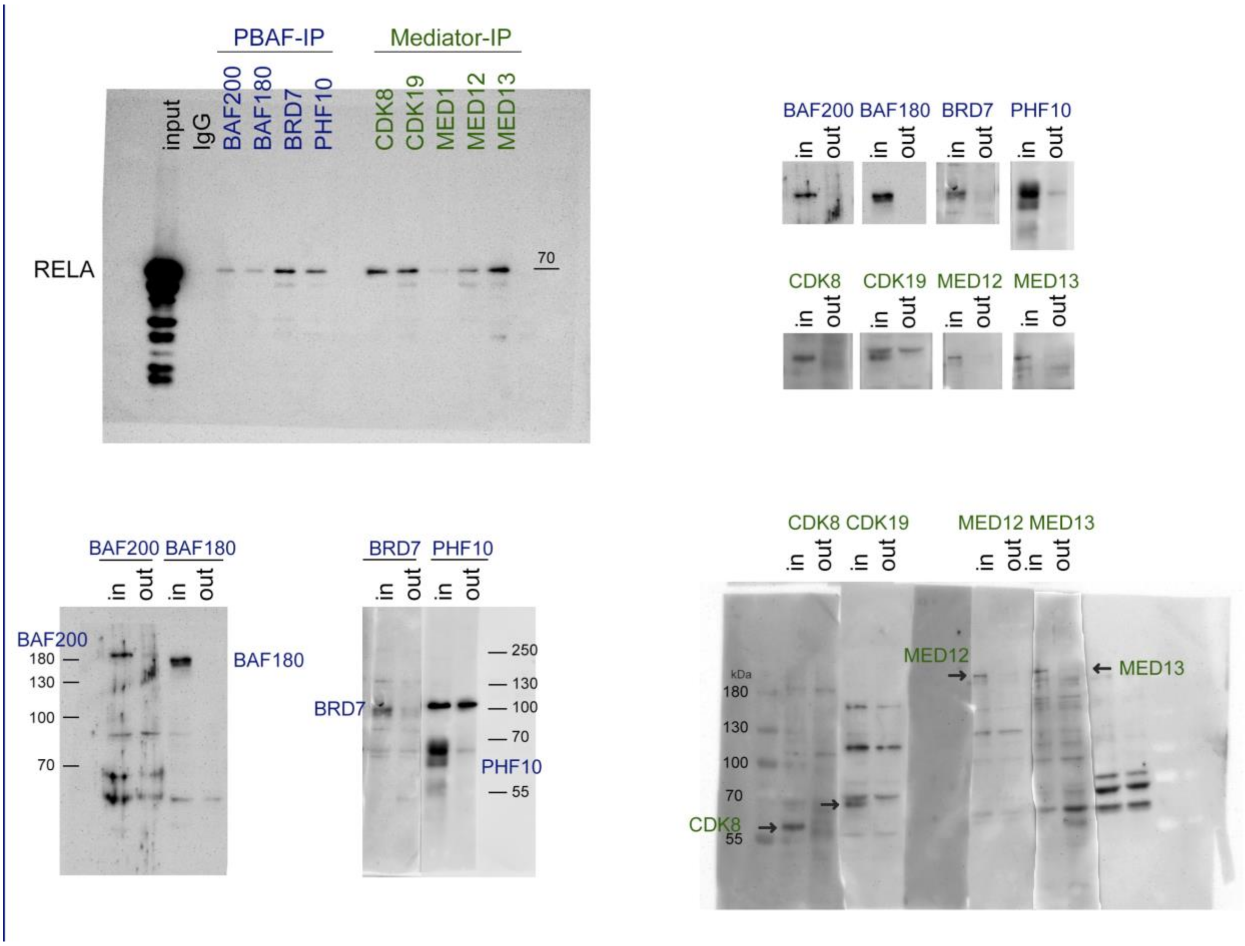
PBAF-IP
Mediator-IP
input
IgG
BAF200
BAF180
BRD7
PHF10
CDK8
CDK19
MED1
MED12
MED13
RELA
70
BAF200 BAF180 BRD7 PHF10
in out
CDK8 CDK19 MED12 MED13
BAF200 BAF180
BAF200
180
130
100
70
BAF180
BRD7 PHF10
BRD7
250
130
100
70
PHF10
55
CDK8 CDK19
MED12 MED13
MED12
MED13
kDa
180
130
100
70
CDK8
55

Fig. 3A

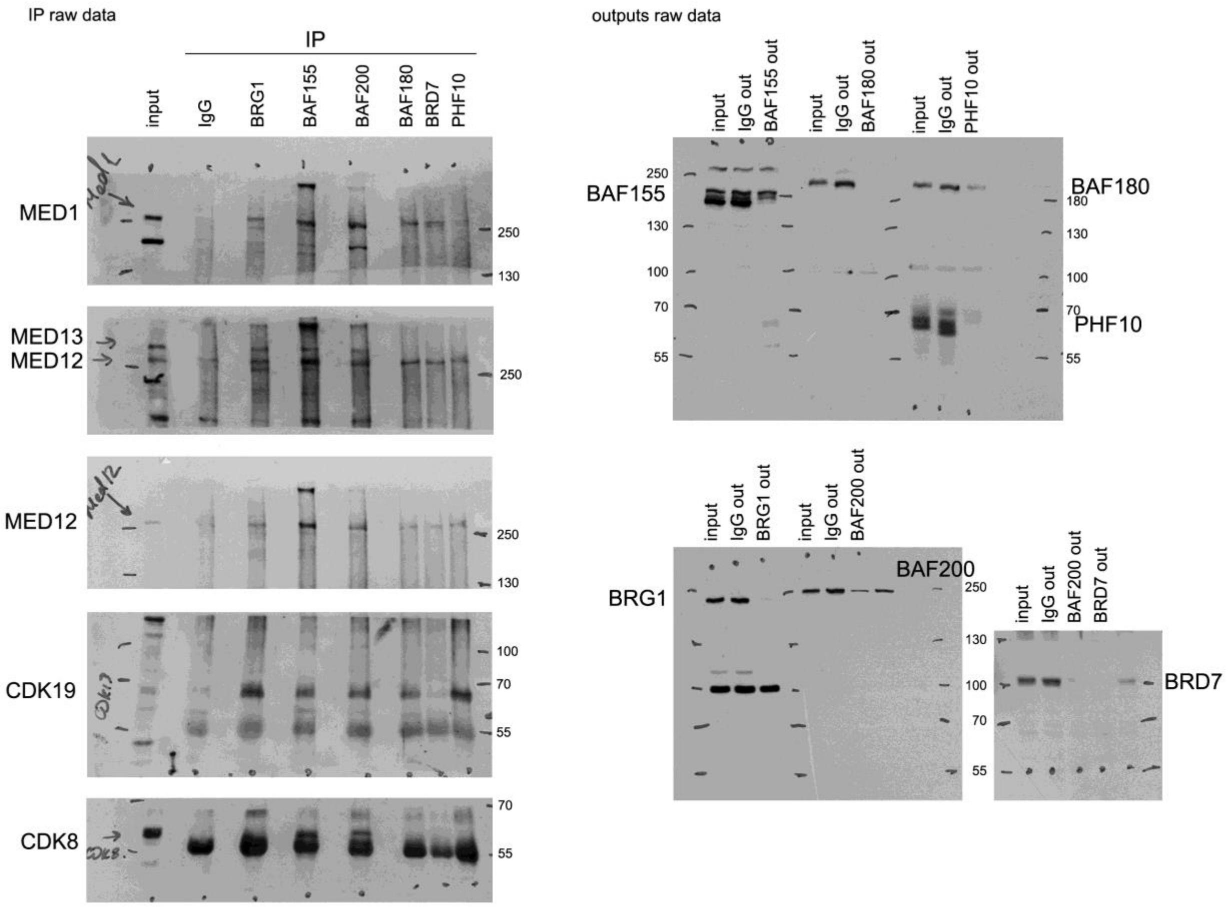
IP raw data
IP
input
IgG
BRG1
BAF155
BAF200
BAF180
BRD7
PHF10
MED1
MED13
MED12
MED12
CDK19
CDK8
250
130
100
70
55
outputs raw data
input
IgG out
BAF155 out
BAF180 out
PHF10 out
BAF155
BAF180
PHF10
180
BRG1 out
BAF200 out
BRD7 out
BRG1
BAF200
BRD7

Fig. 3B

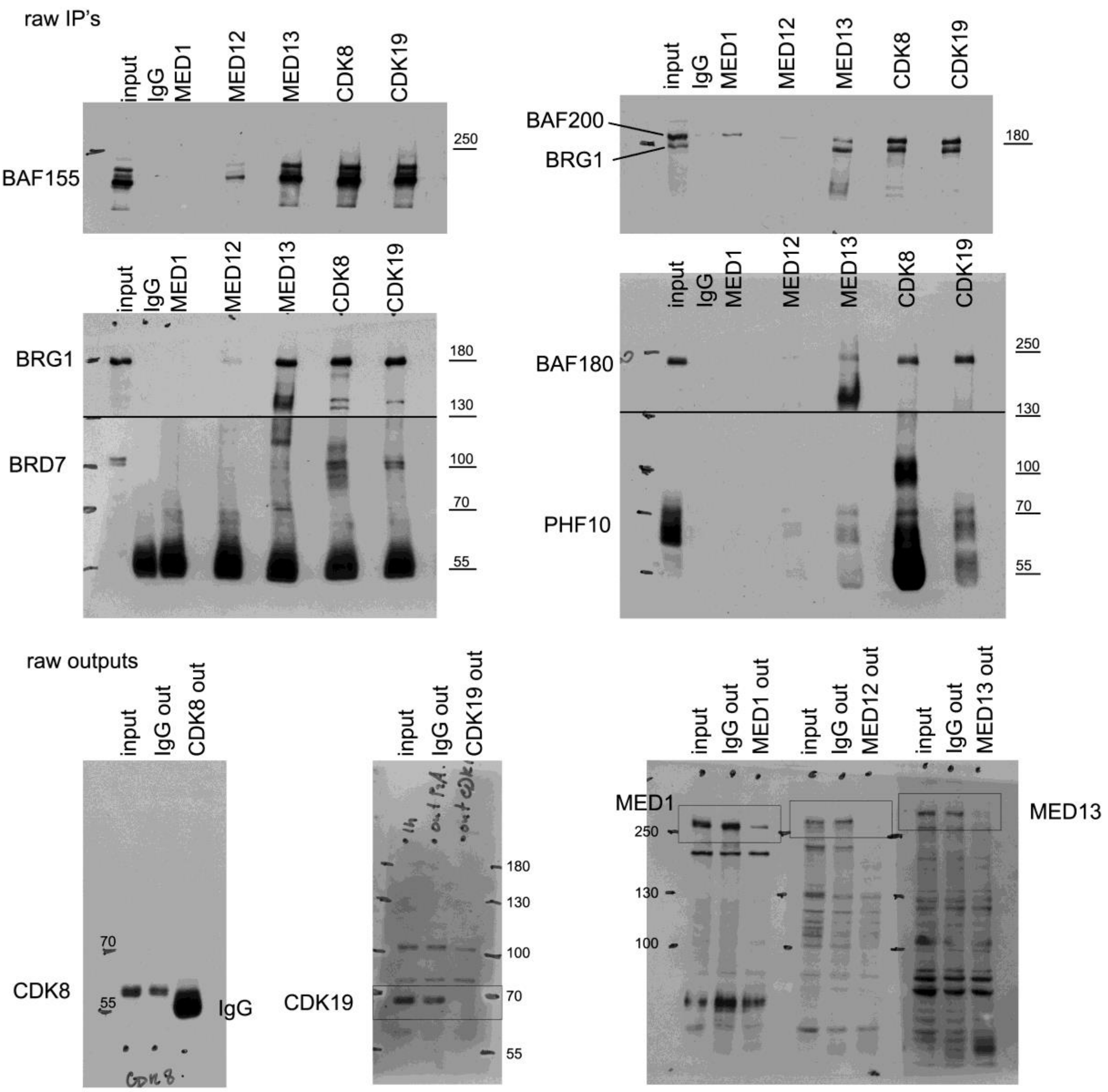
raw IP's
input
IgG
MED1
MED12
MED13
CDK8
CDK19
BAF155
250
BAF200
BRG1
180
BRG1
BRD7
180
130
100
70
55
BAF180
PHF10
250
130
100
70
55
raw outputs
input
IgG out
CDK8 out
CDK8
IgG
70
55
CDK19 out
CDK19
180
130
100
70
55
MED1 out
MED12 out
MED13 out
MED1
MED13
250
130
100
